# Novel potential biomarkers for predicting childhood caries via metagenomic analysis

**DOI:** 10.3389/fcimb.2025.1522970

**Published:** 2025-06-17

**Authors:** Hui Zhang, Xiao Zheng, Yongmao Huang, Yuanqiang Zou, Tao Zhang, Maria Alice Repo, Meixiang Yin, Yang You, Zhuye Jie, Wen-an Xu

**Affiliations:** ^1^ Shenzhen Clinical College of Stomatology, School of Stomatology, Southern Medical University, Shenzhen, Guangdong, China; ^2^ Department of Pediatric Dentistry, Shenzhen Stomatology Hospital (Pingshan), Southern Medical University, Shenzhen, Guangdong, China; ^3^ School of Life Sciences, Fudan University, Shanghai, China; ^4^ Greater Bay Area Institute of Precision Medicine (Guangzhou), Fudan University, Guangzhou, China; ^5^ Beijing Genomics Institute (BGI) Research, Shenzhen, China; ^6^ BGI Research, Wuhan, China; ^7^ Department of Stomatology, Shenzhen Samii Medical Center, Shenzhen, China; ^8^ Department of Stomatology, Zhujiang Hospital, Southern Medical University, Guangzhou, China

**Keywords:** dental caries, oral microbiome, metagenomic sequencing, biomarkers, children

## Abstract

**Background:**

Dental caries is a prevalent global health issue, particularly among children, with significant oral and overall health implications. The oral microbiome is considered a critical factor in caries development, with various microbial species implicated in the disease process.

**Objectives:**

This study aims to explore the changes and interactions of oral microbiota in childhood caries using metagenomic analysis, and identify potential biomarkers for early caries detection and treatment.

**Methods:**

Saliva samples were collected from 241 children aged 6 to 9 years, categorized into caries-free (CF), low-caries (CL), and caries-severe (CS) groups. Metagenomic sequencing was performed to analyze the oral microbiome, followed by a series of statistical and functional analyses to characterize microbial diversity and function.

**Results:**

The study revealed significant differences in the microbial community composition among the groups, with the CS group exhibiting higher alpha and beta diversity than that of the CF group. Numerous unclassified microorganisms, such as *Campylobacter SGB19347* and *Catonella SGB4501*, are intimately linked to dental caries and display intricate interaction networks, suggesting the potential formation of a distinct ecological network. In functional assessment, we identified a possible link between pectin and caries, suggesting that microorganisms that produce pectinase enzymes might play a role in the advancement of severe dental caries. Additionally, we identified 16 species as the best marker for severe dental caries, achieving an impressive AUC of 0.91.

**Conclusion:**

The role of microbiota in dental caries is multifaceted, involving a complex interplay of microbial species and functions. Our findings enhance the understanding of the microbial basis of dental caries and offer potential diagnostic and therapeutic targets. The predictive capacity of the identified biomarkers warrants further investigation for early caries detection and intervention.

**Clinical Significance:**

The identification of novel biomarkers through metagenomic analysis enables early detection and targeted intervention for childhood caries, potentially transforming children dental care and significantly improving long-term oral health outcomes.

## Introduction

Dental caries represent a significant global public health challenge, imposing a substantial burden on health care systems and economies worldwide (Wen et al., 2022). It affects an estimated 514 million children globally (Benzian et al., 2022), with prevalence rates escalating over time and maintaining a high level in mainland China (Gu et al., 2019). Caries is a multifactorial disease and its consequence is the lesion in the hard tissue (Yip and Smales, 2012; Pitts et al., 2017), dental caries not only compromise oral health (Taubman and Nash, 2006), but can also have profound implications for children’s overall physical and mental health in severe cases (Selwitz et al., 2007; Souza et al., 2018).

Dental caries arises not just from poor oral care, a sugary diet and complex interplays of immunological and genetic predispositions, but also from the ecological plaque hypothesis (Freire et al., 2021), which posits that the predominance of acid-producing and acid-tolerant bacteria in dental plaque disrupts the demineralization/remineralization equilibrium (Takahashi and Nyvad, 2011). Acidogenic bacteria, including *Streptococcus mutans* (Welin-Neilands and Svensater, 2007; Forssten et al., 2010; Niu et al., 2020) and *Lactobacillus* (Wen et al., 2022), produce acids within the dental plaque on the teeth surface (Marsh, 2006), leading to a localized decrease in pH (Takahashi, 2015), resulting in a net loss of minerals and the subsequent initiation of dental caries. Despite the growing body of research, the complex interplay between microbial communities and the initiation of dental caries remains an area of active investigation, with findings varying across studies due to diverse methodologies and sampling techniques.

The conventional analysis of 16S rRNA gene sequencing data often treats each taxon as an independent variable (Dueholm et al., 2020), potentially overlooking the complex interactions between microbes that are essential for a comprehensive understanding of dysbiosis. Metagenomic sequencing (SMs) lies in the capacity for performing strain-level taxonomic analysis, enabling the precise reconstruction of microbial strains and the functional annotation with pathway predictions of the studied microbiome (Forster et al., 2019; Integrative, H. M. P. R. N. C, 2019; Han et al., 2020). A 2019 study of 30 children in supragingival microbiomes reported a core bacteriome, including *Prevotella*, *Veillonella*, as yet *unnamed Actinomyces*, and *Atopobium* showed strongest.

association with caries (Al-Hebshi et al., 2019). While a 2022 preliminary study of 40 adolescents highlighted the potential role of microbial communities, including *Lactobacillus*, *Actinomyces*, and *Streptococcus mutans*, in the development of dental caries among varying sugar consumption patterns, the relevance of these findings to a more diverse oral health demographicly remains uncertain due to the limited scope (Pang et al., 2022).

Similarly, a 2023 study involving 51 children in salivary microbiome composition reported that *Prevotella*, *Veillonella*, *Actinomyces*, and *Mogibacterium* were found at significantly elevated concentrations in adolescent caries lesions (Liu et al., 2023). However, due to the small sample size, their conclusions regarding the salivary microbiome changes may not extrapolate to the broader population’s oral microbiome. These studies have investigated the relationship between oral microbiota and dental caries. However, they have not assessed the interactions between differentially abundant species, nor provided an in-depth functional analysis of the microorganisms. Additionally, they failed to evaluate the predictive capacity of these species for disease.

With these advancements in SMs, our study sample size has increased to 241 individuals, aiming to delve deeper into the complex interactions of the oral microbiome, aiming to uncover novel biomarkers and mechanisms associated with dental caries, thereby enhancing our preventive and therapeutic strategies. We conducted a metagenomic sequencing analysis of the oral microbiome to identify markers linked to dental caries, elucidate the interactions among oral microorganisms, and perform comprehensive functional profiling of cariogenic microbes, aiming to develop microbial-based predictive models for dental caries.

## Materials and methods

### Ethics approval

The study received approval from The Ethics Committee of Shenzhen Stomatology Hospital (Pingshan) of Southern Medical University, including the design and consent procedure (reference 202302A). Parental or guardian understanding of the research was ensured and written informed consent was obtained from all of the child participants. The execution of all experiments adhered to the approved guidelines.

### Study population

Subjects in the study were children recruited from 9 primary schools in Pingshan District, Shenzhen from July to September 2023. During the study, a standardized protocol was consistently applied, with all oral examinations conducted by the same dentist. The dentist, trained and experienced in caries assessment, demonstrated a high level of inter-rater reliability, as evidenced by a Kappa value of 0.87, ensuring the consistency and accuracy of the examination process. During the oral examination, both permanent and primary teeth underwent assessment to determine their overall health status. The evaluation of caries followed the standard of the WHO oral health survey basic methods (the 5th edition), including the documentation of the DMFT index and dmft index values. DMFT index is the sum of the number of Decayed, Missing due to caries and Filled teeth in the permanent dentition and dmft index is the sum of the number of decayed, missing due to caries and filled teeth in the primary teeth (Organization, W. H, 2013). Inclusion criteria include: 1) children aged 6–9 years without any history of permanent tooth caries; 2) able to cooperate with the examinations and operations required for the project during the study; 3) voluntary participation in the study; and 4) obtaining informed consent from the guardian. Exclusion criteria included: 1) children with any systemic diseases or drug usage that could influence the oral cavity microbiota or salivary gland functionalities; 2) developmental diseases of teeth; and 3) the usage of antibiotics, probiotics, synbiotics or fluoride three months prior to the study. Children with caries in permanent teeth were excluded from the study, and thus, only the dmft index was used to assess caries status in primary teeth. The grouping criteria consisted of caries-free (CF, dmft = 0), low-caries (CL, 0 < dmft < 6) and caries-severe (CS, dmft ≥ 6). Upon the enforcement of the inclusion/exclusion criteria, a sum of 241 children were recruited for the study. The participants were segmented into three distinct groups: a CF group composed of 48 children, a CL group composed of 99 children and a CS group composed of 94 children.

### Sample collection

For the collection of unstimulated saliva, participants were instructed to allow saliva to accumulate in their mouths for at least one minute. Subsequently, they were asked to let the saliva flow into a pre-labelled 5 mL collection tube (ZOSEN, TZ, Jiangsu, China). This procedure could be repeated multiple times in order to collect larger volumes of saliva (2–5 mL). Following collection, all tubes were promptly positioned in the freezer compartment of a foam incubator and stored at a temperature of -80^°C^ within two hours until further processing.

### DNA extraction, sequencing, and quality control

The extraction of DNA from the samples was conducted employing the MagPure Stool DNA KF Kit B (MD5115, Magen), utilizing 1mL of each sample (Yang et al., 2020). SMs was performed on the MGISEQ-T7 platform (BGI, Shenzhen, China), generating 100 base pair (bp) paired-end reads for all samples. Four libraries were constructed for each lane. The data was subsequently processed with reads being filtered and trimmed using fastp v0.19.4 (Chen et al., 2018), based on stringent criteria- a minimum average quality Phred score of 20, and reads of length not shorter than 51 bp were retained, and host sequence contamination was identified and removed using Bowtie2 v2.3.5 (Langmead and Salzberg, 2012) (human genome GRCh38) and seqtk v1.3 (Shen et al., 2016).

### Data processing

For community composition analysis and procuring the relative bacterial abundances in each sample, MetaPhlAn version 4.0.1 was used with default parameters (Blanco-Miguez et al., 2023). Analysis of microbial gene families and metabolic pathways was performed using HUMAnN3 (v3.0.1) (Franzosa et al., 2018), attributed to the UniRef90 EC filtered database (uniref90_v269_201901) (Suzek et al., 2007), making mapping to the Kyoto Encyclopedia of Genes and Genomes (KEGG) (Ogata et al., 1999) and MetaCyc (Caspi et al., 2006) databases feasible. The data were normalized by Count Per Million (CPM) measures. The final amalgamated results were compiled into a single file using the HUMAnN_join_tables utility.

### Alpha and beta diversity

The alpha diversity of the microbiome among groups was evaluated based on both Shannon and Simpson indices using the ‘diversity’ function in the vegan R package (version 2.6.1). In human oral microbiomes, Shannon and Simpson indices measure microbial diversity. High values of Shannon indices indicate higher species richness and evenness. High values of Simpson index indicate higher diversity of the community. The Shannon index focuses on the richness of the community and rare species, while the Simpson index focuses on uniformity and dominant species in the community. To extrapolate distinctions in community compositions (beta diversity) among the groups, Bray-Curtis distance metrics were computed and a Permutational Multivariate Analysis of Variance (PERMANOVA) with 999 permutations was conducted using the ‘adonis2’ function in the Vegan R package.

### Differential relative abundance enrichment analysis

The significance of variations in relative abundance was scrutinized on a per-species basis using the Wilcoxon test, computed in the tidyverse R package (version 2.0.0). In order to account for multiple hypothesis testing, *p*-values were adjusted using the Bonferroni correction to manage the False Discovery Rate (FDR). The area under the Receiver Operating Characteristic (ROC) curve (AUC) was applied as a non-parametric measure of effect size, calculated for each species using the pROC R package. Additionally, we devised a generalization of the fold change that is broadly applicable to different types of read abundance data. This generalized fold change was derived as the mean difference between the distributions of two groups. Such analyses enabled comparisons between the CL and CF as well as CS and CF groups.

### Co-occurrence correlation network analysis

Co-occurrence correlation networks offer insights into multi-partner microbial interactions. To illustrate these networks in samples from caries-free and severe caries conditions, the Spearman correlations of relative abundances for all microbial species pairs were computed. The resulting *p*-values underwent an adjustment through the Benjamini-Hochberg correction. Connections, either positive or negative, were established between species pairs exhibiting adjusted *p*-values lesser than 0.0005. These networks were then visualized using the Cytoscape software (version 3.9.1).

### Functional profiling

To determine the significance of variations in relative abundance, each pathway was examined using the Wilcoxon test, as provided in the tidyverse R package (version 2.0.0). To account for multiple hypothesis testing, *p*-values were adjusted using the Bonferroni correction, in order to acknowledge the FDR. A comparative evaluation of the KEGG pathways and MetaCyc pathways was performed to discern the distinct microbial metabolic pathways between the CS and CF groups. We identified differentially expressed metabolic pathways with statistical significance, including those from the KEGG pathways (FDR-adjusted *p*-values < 0.01) and the MetaCyc pathways (FDR-adjusted *p*-values < 0.2). As a result, a selection of two KEGG pathways and seven MetaCyc pathways was identified as significantly differentially expressed. Next, in order to observe the expression of these selected pathways in the three groups, we used the ‘stat_compare_means’ function in the ggpubr R package and Wilcoxon test to compare the pathways of three groups.

### Establishment of severe caries prediction model

The establishment of a severe caries prediction model was achieved through the use of Recursive Feature Elimination (RFE). This algorithm involves iterative modeling for selecting features. Each iteration secures the top-ranked predictors, reevaluates the model, and determines the optimal model based on the highest accuracy. The ‘rfe’ function in the caret R package was employed for this analysis with specified parameters: functions= ‘newRF’, method = ‘repeatedcv’, metric = ‘ROC’, and ntree=1000. Subsequently, our focus shifted towards the species exhibiting the highest predictive potential for distinguishing between the CF and CS groups. For the comparison of these species across three groups, we employed the “stat_compare_means” function in the ggpubr R package and the Wilcoxon test.

## Results

### Sample collection and sequencing features

In this study, we investigated the saliva microbiota composition in 241 primary school children aged 6 to 9 years using SMs. The children were categorized into three groups based on their dental caries status: caries-free (CF, dmft = 0, n = 48), low-caries (CL, 0 < dmft < 6, n = 99), and caries-severe (CS, dmft ≥ 6, n = 94), where ‘dmft’ represents the sum of decayed, missing (due to caries), and filled teeth in primary teeth. The participants in each group were matched in terms of age and gender, and their demographic and clinical information can be found in **Supplementary Table 1**. Non-stimulated saliva samples were collected from the children for further analysis of the oral microbiota. After conducting shotgun sequencing, data were obtained for 241 samples, with an average sequencing data of 22.64 ± 0.78 Gb per sample. Upon completion of the quality control assessment, each sample produced an average of 147.09 million initial paired reads. Following the elimination of host reads, 35.17 million clean paired reads were obtained per sample.

### Caries-induced alterations in the oral microbiome

**Figure 1:**
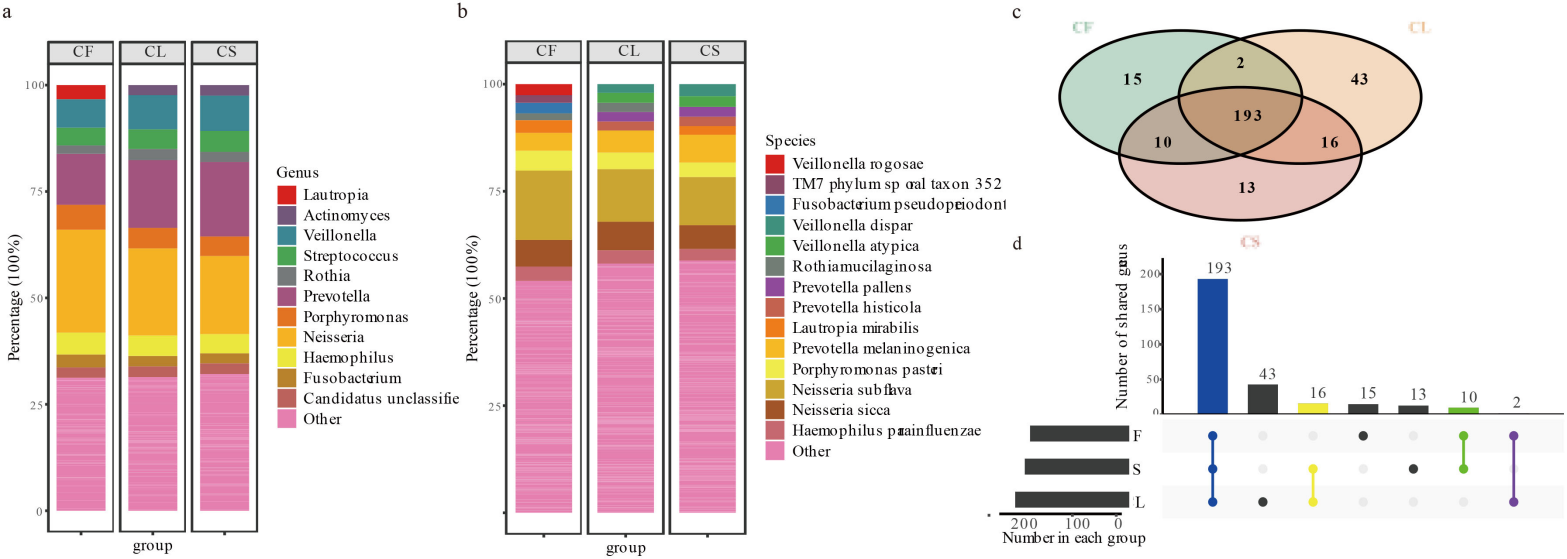
Description of taxa between CF, CL and CS group at each taxonomic level. **(a)** the microbial composition analysis of the CF, CL and CS group at the genus. **(b)** the microbial composition analysis of the CF, CL and CS group at the species. **(c)** the collection of the CF, CL and CS group in the genus. **(d)** the upset plot of the CF, CL and CS group in the genus.

In this study, we identified a diverse array of bacterial and archaeal taxa using MetaPhlAn4, comprising a total of 2 kingdoms, 15 phyla, 60 classes, 83 orders, 115 families, 292 genera, and 744 species. The oral microbiota of children was dominated by several core genera, with *Neisseria* being the most prevalent at 20.37%. Other significant genera included *Prevotella* (15.74%), *Veillonella* (7.86%), *Porphyromonas* (4.94%), *Haemophilus* (4.76%), *Streptococcus* (4.65%), *Fusobacterium* (2.52%), *Candidatus Saccharibacteria unclassified* (2.51%), *Actinomyces* (2.32%), and *Rothia* (2.31%; **Figures 1a, b**). Together, these genera accounted for approximately 68.77% of the oral microbiota in the CF group, 68.59% in the CL group, and 67.88% in the CS group. A total of 193 genera were shared among the three groups. Additionally, two genera were shared between the CF and CL groups, 10 genera were shared between the CF and CS groups, and 16 genera were shared between the CL and CS groups. Furthermore, 13 genera were exclusive to the CS group, 15 genera were exclusive to the CF group, and 43 genera were exclusive to the CL group (**Figures 1c, d**) (**Supplementary Table 4**).

**Figure 2:**
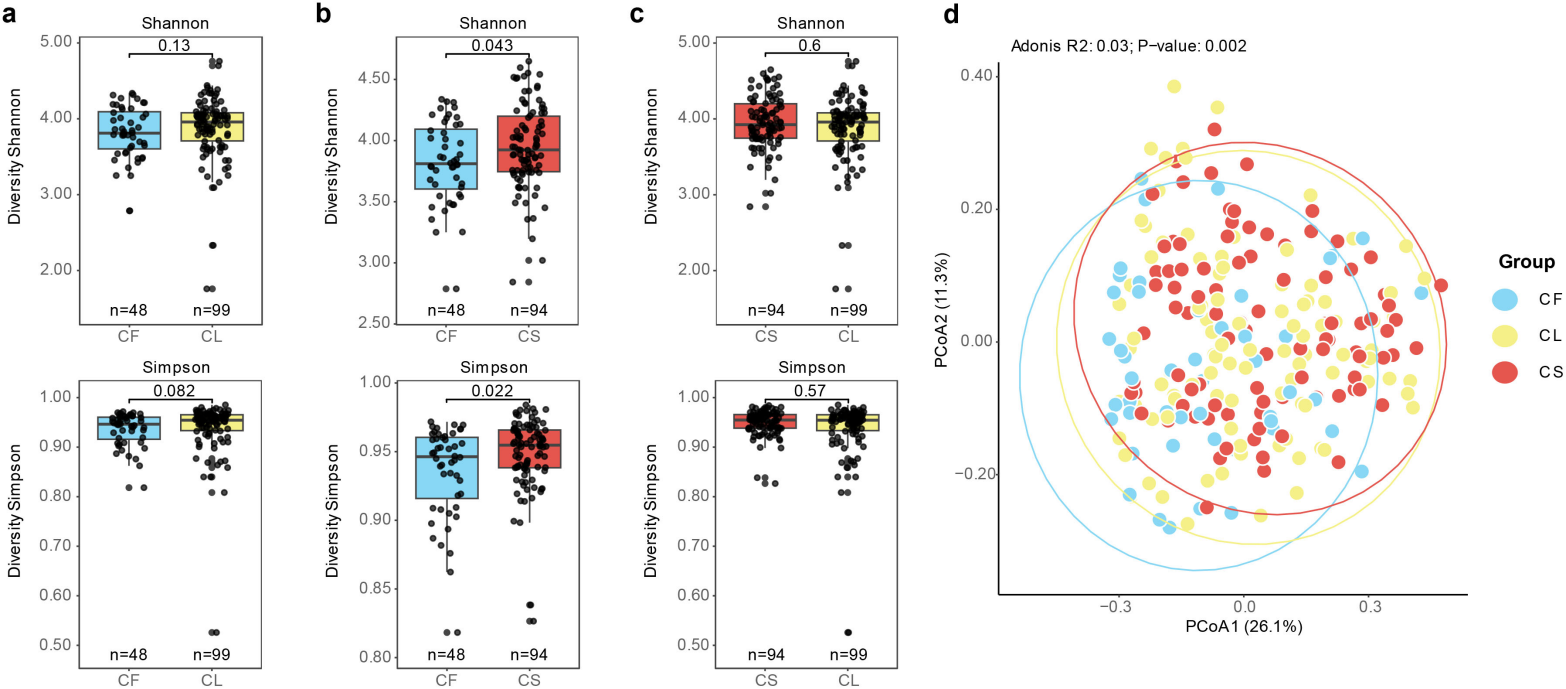
Association between microbiota composition and caries status. **(a)** Shannon and Simpson index between the CF group and the CL group (*p* = 0.13, *p* = 0.082). **(b)** Shannon and Simpson index between the CF group and the CS group (*p* = 0.043, *p* = 0.022). **(c)** Shannon and Simpson index between the CL group and the CS group (*p* = 0.6, *p* = 0.57). **(d)** PCoA analysis of the CF, CL and CS group based on Bray-Curtis distance between the bacterial communities present in all specimens (R^2 ^= 0.03%, *p* = 0.001).

Next, we conducted α-diversity analysis comparing the values of Shannon and Simpson indices among different groups. We observed that the CF group exhibited lower diversity compared to the CS group (**Figure 2b**). No significant differences were detected in terms of Simpson and Shannon indices between the CF and CL groups, as well as between the CL and CS groups (**Figures 2a, c**). Furthermore, we performed principal co-ordinates analysis (PCoA) based on the Bray-Curtis dissimilarity index to evaluate the compositional differences among the different groups (**Figure 2d**). We observed significant differences in the microbial communities among these three groups, as determined by adonis analysis (R^2^ = 0.03%, *p* = 0.001). *Post-hoc* analysis, we found significant microbial composition differences between the CF and CL groups (*p* = 0.003), as well as between the CF and CS groups (*p* = 0.003) (**Supplementary Table 2**). These findings indicated that microbial structure was associated with the development and progression of dental caries.

### Biomarkers and interactions in the caries-associated microbiota

**Figure 3:**
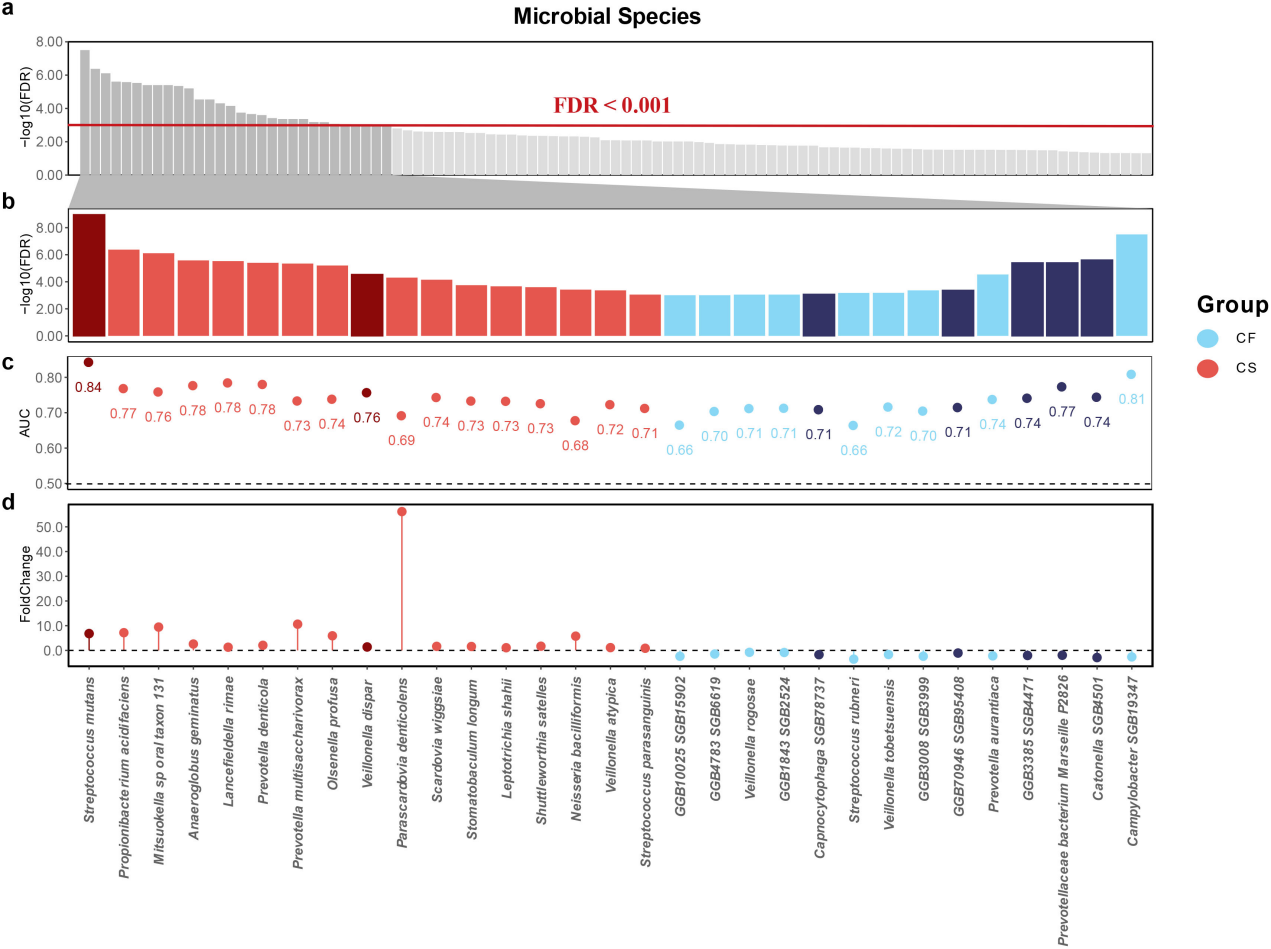
Potential salivary microbial biomarkers associated with severe caries. **(a)** microbial biomarkers associated with severe caries in the saliva samples at FDR < 0.001. **(b)** thirty-one species were observed to be closely associated with severe caries and their FDR values ​​were taken and ranked. Red sectors represent for severe caries associations and blue sectors represent for caries-free associations. Dark red and dark blue represent the first expression pattern, and light red and light blue represent the second expression pattern **(c)** the AUC index of thirty-one species. **(d)** the Foldchange index of thirty-one species.

Next, we employed the Wilcoxon test to identify caries-associated microbial species. Compared to the CF group, we identified a number of biomarkers in the CL group, and an even larger number were observed in the CS group. We observed two distinct patterns of differential species enrichment with statistical significance (*p* < 0.0005, FDR < 0.001; **Figure 3a**): The first pattern showed an increase in differential enrichment from the non-carious to the initial caries stage and further to the severe caries stage, while the second pattern was characterized by enrichment exclusive to the severe caries stage.

The former was characterized by: *Streptococcus mutans* (*p* = 1.64 × 10^−12^, FDR = 7.49 × 10^−10^) and *Veillonella atypica* (*p* = 1.48 × 10^−5^, FDR = 2.93 × 10^–4^) were significantly elevated, while *Prevotellaceae bacterium Marseille P2826* (*p* = 5.93 × 10^−8^, FDR = 2.70 × 10^−6^), *GGB3385 SGB4471* (*p* = 5.73 × 10^−8^, FDR = 2.70  × 10^−6^), *GGB70946 SGB95408* (*p* = 1.42 × 10^−5^, FDR = 2.93 × 10^−4^), *Capnocytophaga SGB78737* (*p* = 3.28 × 10^−5^, FDR = 5.75 × 10^−4^), and *Catonella SGB4501* (*p* = 6.15 × 10^−13^, FDR = 1.67 × 10^−6^) were depleted. The latter pattern was destructive lesion 24 species that showed differential enrichment exclusively in the severe caries group, including *Propionibacterium acidifaciens* (*p* = 1.88 × 10^−9^, FDR = 2.86 × 10^−7^), *Mitsuokella* sp *oral taxon 131* (*p* = 4.61 × 10^−9^, FDR = 5.26 × 10^−7^), *Anaeroglobus geminatus* (*p* = 2.35 × 10^−8^, FDR = 1.79 × 10^−6^), *Lancefieldella rimae* (*p* = 3.09 × 10^−8^, FDR = 2.01 × 10^−6^), *Prevotella denticola* (*p* = 5.15 × 10^−8^, FDR = 2.70 × 10^−6^), *Prevotella multisaccharivorax* (*p* = 7.37 × 10^−8^, FDR = 3.06 × 10^−6^), *Olsenella profusa* (*p* = 1.12 × 10^−7^, FDR = 4.26 × 10^−6^), *Veillonella dispar* (*p* = 6.04 × 10^−7^, FDR = 1.97 × 10^−5^), *Parascardovia denticolens* (*p* = 1.10 × 10^−6^, FDR = 3.35 × 10^−5^), *Scardovia wiggsiae* (*p* = 1.68 × 10^−6^, FDR = 4.78 × 10^−5^), *Stomatobaculum longum* (*p* = 4.52 × 10^−6^, FDR = 1.21 × 10^−4^), *Leptotrichia shahii* (*p* = 5.81 × 10^−6^, FDR = 1.47 × 10^−4^), *Shuttleworthia satelles* (*p* = 7.08 × 10^−6^, FDR = 1.70 × 10^−4^), *Neisseria bacilliformis* (*p* = 1.13 × 10^−5^, FDR = 2.58 × 10^−4^) and *Streptococcus parasanguinis* (*p* = 3.70 × 10^−5^, FDR = 6.02 × 10^−4^) were significantly elevated, while *Campylobacter SGB19347* (*p* = 9.38 × 10^−11^, FDR = 2.14 × 10^−8^), *Prevotella aurantiaca* (*p* = 5.75 × 10^−7^, FDR = 1.97× 10^−5^), *GGB3008 SGB3999* (*p* = 1.43 × 10^−5^, FDR = 2.93 × 10^−4^), *Veillonella tobetsuensis* (*p* = 2.34 × 10^−5^, FDR = 4.45 × 10^−4^), *Streptococcus rubneri* (*p* = 2.49 × 10^−5^, FDR = 4.53 × 10^−4^) (Huch et al., 2013; Wang et al., 2017), *GGB1843 SGB2524* (*p* = 3.57 × 10^−5^, FDR = 6.02 × 10^−4^), *Veillonella rogosae* (*p* = 3.85 × 10^−5^, FDR = 6.05 × 10^−4^), *GGB10025 SGB15902* (*p* = 4.55 × 10^−5^, FDR = 6.71 × 10^−4^) and *GGB4783 SGB6619* (*p* = 4.56 × 10^−5^, FDR = 6.71 × 10^−4^) were depleted. In addition, we identified species newly associated with severe caries, of which *Campylobacter SGB19347*, *Catonella SGB4501*, *GGB3385 SGB4471*, *Prevotellaceae bacterium Marseille P2826*, *GGB70946 SGB95408*, *GGB3008 SGB3999*, *Capnocytophaga SGB78737*, *GGB1843 SGB2524*, *GGB10025 SGB15902* and *GGB4783 SGB6619* were significantly depleted in the CS group (**Figures 3b, d**).

**Figure 4:**
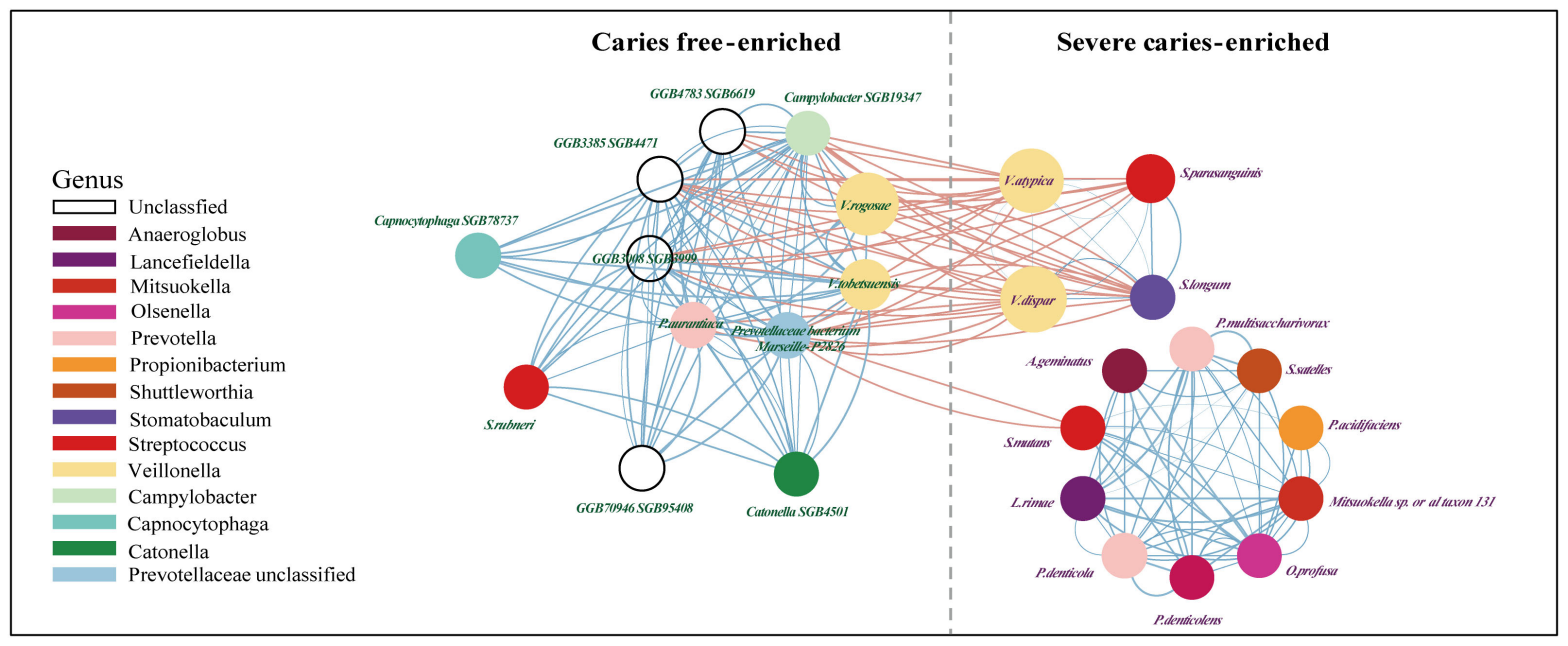
The network between potential salivary microbial biomarkers (value≥0.5). Shows communities (namely, right for caries-free and left for Severe caries) of bacterial species and their positive (blue Pearson coefficient) or negative (red Pearson coefficient) abundances correlation. Alternatively, edge thickness holds an inverse relationship to the Pearson *p*-value and is color-coded based on the Pearson coefficient, with blue representing positive correlations and red, negative ones. A dashed line delineates a “structural gap”, indicating a significant number of negative Pearson correlation edges between caries free and severe caries communities.

Next, a co-occurrence network on these markers was generated to assess potential relationships between the severe caries-associated bacteria. In line with previous studies (Gross et al., 2010; Garcia et al., 2021), some types of acid-producers, from *Streptococcus mutans* to *Propionibacterium acidifaciens*, showed positive correlations with each other but little correlation with the caries-free-enriched bacteria, which may indicate an independent synergistic relationship between these cariogenic microorganisms (**Figure 4**). In addition, bacteria in dental plaque produce acids, leading to a decrease in plaque pH. This acidic environment affects the solubility of hydroxyapatite, initiating the demineralization process (Mosaddad et al., 2019). Our findings show a strong association between acid-producing bacteria, such as *Streptococcus mutans* and *Propionibacterium acidifaciens*, and microorganisms that facilitate dental plaque formation, such as *Anaeroglobus geminatus* (Bao et al., 2017) and *Prevotella denticola* (Niu et al., 2023). An intriguing finding was the distinct distribution of *Streptococcus rubneri*. While other *Streptococcus* species on the co-occurrence network were enriched in the disease group, *Streptococcus rubneri* was significantly enriched in the caries-free samples. This bacterium was distinctly separated from other species of the *Streptococcus* genus in the network analysis and showed a significant synergistic relationship with bacteria from the CF group, such as *GGB3385 SGB4471* and *Catonella SGB4501*, predominantly belonging to the *Lachnospiraceae* family.

These data suggested that oral bacteria significantly influence childhood caries and reveal interactions among various oral microorganisms. This implies that a multitude of currently unidentified microorganisms could potentially play a crucial role in the development of childhood caries.

### Functional metagenomic signatures between severe caries and caries free

Unlike 16S rRNA gene amplicon data, shotgun metagenomics data facilitates a direct analysis of the microbiome’s functional potential. Using this approach, we examined orthologous gene family’s difference in abundance between the CS and CF groups by HUMAnN3 software. We identified some new genes associated with caries.

**Figure 5:**
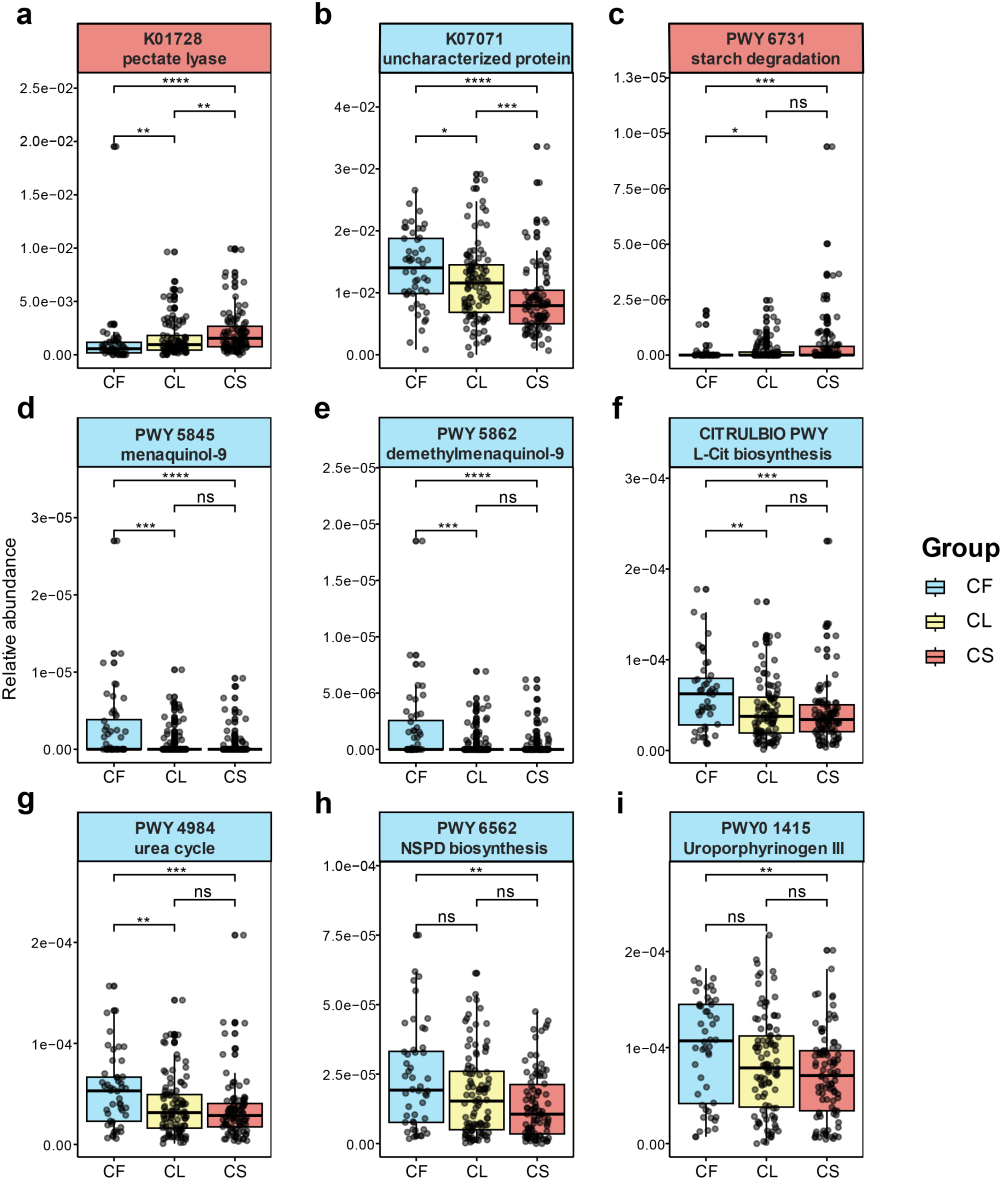
Functional metagenomic signatures between the CS and CF group. differentially expressed KEGG pathways between the CS and CF group **(a, b)**. **(a)** pectate lyase (K01728). **(b)** uncharacterized protein (K07071). differentially expressed Metacyc pathways between the CS and CF group **(c-i)**. **(c)** the starch degradation III pathway (PWY-6731). **(d)** the superpathway of menaquinol-9 biosynthesis (PWY-5845). **(e)** the superpathway of demethylmenaquinol-9 biosynthesis (PWY-5862). **(f)** L-citrulline (L-cit) biosynthesis (CITRULBIO-PWY). **(g)** urea cycle (PWY-4984). **(h)** norspermidine (NSPD) biosynthesis (PWY-6562). **(i)** uroporphyrinogen-III (PWY0-1415). (*p < 0.05, **p < 0.01, ***p < 0.001, ****p < 0.0001).

In the Kyoto Encyclopedia of Genes and Genomes (KEGG) pathways, pectate lyase (K01728, *p *= 6.44 × 10^−7^, FDR = 0.00397) showed significantly elevation in the CS group (**Figure 5a**), and the species from *Leptotrichia* and *Pectobacterium* (Tarasova et al., 2013; Kuilenburg et al., 2016), including *Leptotrichia* sp *oral taxon 498* (*p* = 1.80 × 10^−4^, *FDR* = 0.00201) and *Leptotrichia wadei* (*p* = 0.0162, *FDR* = 0.00201) were identified as species closely containing pectate lyase. While uncharacterized protein (K07071, *p *= 6.84 × 10^−7^, FDR = 0.00397) elevated in the CF group (**Figure 5b**).

In the Metabolic Pathways From all Domains of Life (MetaCyc) pathways, genes involved in starch degradation (PWY-6731, *p* = 9.39 × 10^−4^, FDR = 0.08884) (**Figure 5c**), which were involved in bacterial acid production (Reddy et al., 2008; Abedi and Hashemi, 2020) were predominantly elevated in the CS group. Genes associated with the biosynthesis of menaquinol-9 (PWY-5845, *p* = 9.63 × 10^−5^, FDR = 0.02278) (**Figure 5d**) and demethylmenaquinol-9 (PWY-5862, *p* = 9.63 × 10^−5^, FDR = 0.02278) (**Figure 5e**) were significantly elevated in the CF group. Additionally, pathways for L-citrulline (L-Cit) biosynthesis (CITRULBIO-PWY, *p* = 4.40 × 10^−4^, FDR = 0.0693) (**Figure 5f**), urea cycle (PWY-4984, *p* = 6.31 × 10^−4^, FDR = 0.0746) (**Figure 5g**), superpathway of heme b biosynthesis from uroporphyrinogen-III (PWY0-1415, *p *= 0.00156, FDR = 0.1054) (**Figure 5i**) and norspermidine (NSPD) biosynthesis (PWY-6562, *p* = 0.00148, FDR = 0.1054) (**Figure 5h**), which were responsible for production of arginine (Nascimento, 2018), were significantly elevated in the CF group. The species from *Lautropia* and *Klebsiella*, such as *Lautropia mirabilis* (*p* = 0.00278, FDR = 0.01691), were identified as being containing arginine anabolism.

Overall, caries-associated microorganisms are closely related to carbohydrate metabolism, while non-caries-associated microorganisms are closely related to arginine anabolism.

### Saliva microbiota as a potential diagnostic biomarker for severe caries

Most cases of dental caries can be prevented through effective plaque control, and there is a significant opportunity for early detection with regular check-ups (Rodrigues et al., 2022).

**Figure 6:**
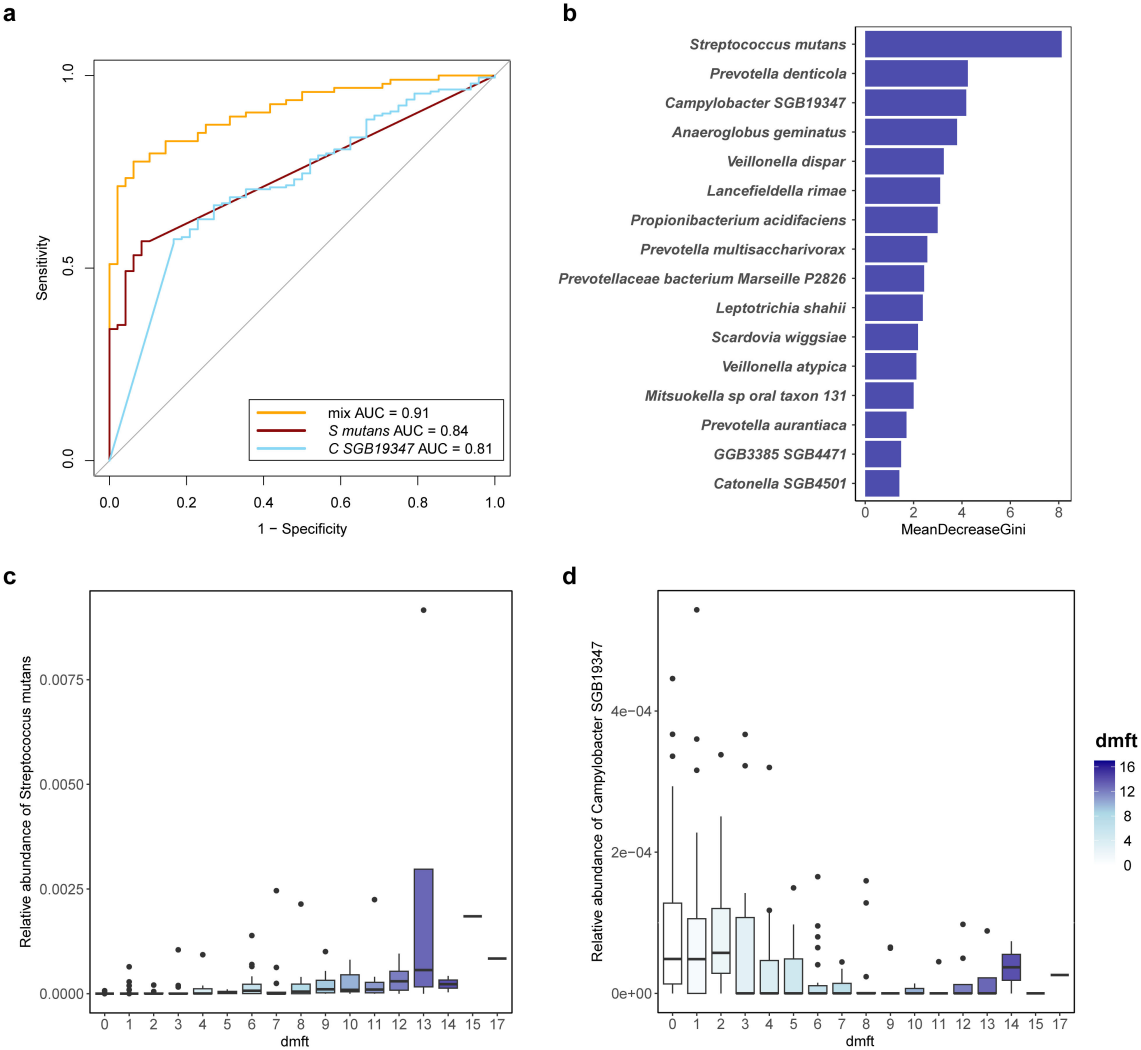
Potential diagnostic biomarker for severe caries. **(a)** consortia of bacteria predict severe caries status. Receiver Operating Characteristic (ROC) with tenfold cross-validation was employed to assess the best oral bacterial species, and the best species for predicting severe caries and its AUC value are shown. orange line represents AUC of the top 16 species-level predictors, dark red line represents AUC of the top severe caries enriched predictive species (*Streptococcus mutans, S mutans*) and blue line represent AUC of the top severe caries enriched predictive species (*Campylobacter SGB19347, C SGB19347*). **(b)** the top 16 species-level predictors (determined by mean decrease GINI) for a random forest model predicting severe caries. **(c)** variation in relative abundance of the top severe caries enriched predictive species (*Streptococcus mutans, S mutans*) with dmft index. **(d)** variation in relative abundance of the top caries-free enriched predictive species (*Campylobacter SGB19347, C SGB19347*) with dmft index.

Having characterized the differences in microbiota composition between children in different caries state and those in a caries-free state, we sought to understand the ability of discriminate children with severe caries from healthy children and constructed random-forest classifiers. When using a single species to build a model, the random forest classifiers achieved an AUC ranging from 0.66 to 0.84 for detecting patients with severe caries (**Figure 3c**). *Streptococcus mutans* was the top severe caries enriched predictive species, with an AUC of 0.84, and its relative abundance gradually increased with the dmft index (**Figure 6c**). *Campylobacter SGB19347* was the top caries free enriched predictive species, with an AUC of 0.81. Interestingly, the relative abundance of *Campylobacter SGB19347* was significantly elevated in the CF and CS groups compared to the CL group, where it exhibited a notable decrease (**Figure 6d**).

Next, we established a prognostic risk model for severe caries through a recursive feature elimination (RFE) model for selecting the best sets of biomarkers. The RFE algorithm with 10-folds cross-validation identified 16 species as the best marker set for severe caries, based on feature importance in the model. These included *Streptococcus mutans, Prevotella denticola, Campylobacter SGB19347, Anaeroglobus geminatus, Veillonella dispar*, *Lancefieldella rimae, Propionibacterium acidifaciens, Prevotella multisaccharivorax, Prevotellaceae bacterium Marseille P2826*, and others (**Figure 6a**). These species demonstrated an AUC of 0.905 within the RFE model, indicating strong predictive performance for severe caries.

All 16 species in the optimal set were previously identified as screened biomarkers (**Figure 6b**), indicating that they can offer enhanced diagnostic information for detecting severe dental caries based on the salivary microbiome. Consequently, these species can be considered as potential biomarkers for severe dental caries.

## Discussion

Over the last 23 years (Li et al., 2023), microbiome research has transitioned from culture-based methods to sophisticated molecular profiling (Villmones et al., 2022), significantly enhancing our understanding of human microbiota across various body niches (Ye et al., 2019). To fully comprehend the connection between oral microorganisms and diseases, future studies must integrate metagenomics and expand sample sizes for more robust findings (Pasolli et al., 2019; Casals-Pascual et al., 2020). In our research, which boasted a larger sample size relative to previous researches (**Supplementary Table 3**), we performed a metagenomic analysis on saliva samples from children to explore the relationship between microbes and caries. Our study reveals significant taxonomic and functional differences in the microbiomes of caries-free children and those with different levels of caries severity. Acid-producing bacteria, such as *Streptococcus mutans* and *Propionibacterium acidifaciens*, elevated in the CS group, while some unnamed bacteria, such as *Campylobacter SGB19347* and *Catonella SGB4501* elevated in the CF group. These results suggest that dental caries could influence the diversity of microbial species in saliva. Furthermore, our analysis revealed a significant association between caries and a variety of microorganisms, including numerous unidentified species in addition to the known ones.

Initially, we compared the differences in microbial composition at the genus level across the CF, CL and CS groups. Compared with the CF and CS groups, the CL group showed more exclusive genera. In the low stages of caries, ecological imbalance may occur, allowing diverse bacterial taxa to proliferate as the community transitions from a healthy state. This may explain the higher number of unique genera in the CL group. However, as caries progresses to a severe state, the microbial community may become dominated by a few highly competitive and acid-tolerant cariogenic bacteria. This dominance could suppress the growth of other taxa, resulting in a reduction in the number of unique genera.

Furthermore, we contrasted the variances in microbial communities and discovered significant statistical differences in the alpha diversity between the caries group and the healthy group. Both Shannon and Simpson indices were noted to be higher in the severe caries group, suggesting that severe caries may enhance the diversity of oral microorganisms. Previous studies present conflicting reports on oral microbial community diversity in dental caries, with some indicating a decrease and others suggesting no significant change in the diversity of oral microbial communities (Jiang et al., 2019). As caries progresses, the oral environment undergoes significant changes, such as pH fluctuations and nutrient availability, which may create niches for diverse microbial taxa to thrive (Takahashi and Nyvad, 2008). This dynamic shift could explain the higher diversity observed in severe caries, despite the dominance of acid-tolerant species. We hypothesize that the variation may be attributed to distinct sequencing approaches, such as our application of metagenomic sequencing and Metaphlan4 in our study, which indicate that the choice of sequencing methods and depth of sequencing may influence the evaluation of the association between dental caries and oral microorganisms.

Subsequently, potential biomarkers distinguishing the various groups. Seven species exhibited distinctions (FDR < 0.1) in the comparison between the CL group and the CF group, and these same species also showed discrepancies in the comparison between the CS group and the CF group. Particularly noteworthy were nine unnamed differentially expressed species in the healthy group (FDR < 0.001) in the comparison between the severe-caries group and the healthy group, which have not been previously linked to caries. *Campylobacter SGB19347* is a distinct species within the CF group. Comparison with other Campylobacter species revealed a correlation with the anabolic metabolism of arginine, leading to a beneficial impact on oral microecology (Zheng et al., 2017). This includes inhibiting cariogenic microorganisms and promoting remineralization of demineralized teeth surfaces (Koopman et al., 2015; Bijle et al., 2018; Jing et al., 2022; Yip et al., 2023). Furthermore, *Prevotellaceae bacterium Marseille P2826*, which appeared as a significantly distinct species in the healthy group, revealed a mutually synergistic relationship with *Campylobacter SGB19347*. The *Prevotella* genus ranked as the second most prevalent genus in both oral sites, consistent with prior research (Tett et al., 2021). However, this particular bacterium remains relatively underexplored and has, to date, shown correlation solely with taste perception (Licandro et al., 2023). Our correlation analysis revealed a mutually inhibitory relationship between *Prevotellaceae bacterium Marseille P2826* and *Streptococcus mutans*. Specifically, *Streptococcus mutans* appears to inhibit the former, suggesting a unidirectional relationship.

Within the severe caries group, *Streptococcus mutans* is prominently expressed and recognized as a principal etiological factor in caries development, a role underscored by its significant contribution to the disease process (Bowen and Koo, 2011; Krzysciak et al., 2014; Lemos et al., 2019). Moreover, *Propionibacterium acidifaciens*, initially detected in human carious lesions by Xiao et al (Xiao et al., 2013), is noted for its acid-producing and acid-resistant properties. Aciaca *Anaeroglobus geminatus*, closely linked to oral biofilms, assists in facilitating the adherence of other species (Bao et al., 2017), thereby facilitating biofilm maturation (Shao et al., 2023). Our correlation analysis has unveiled a robust relationship between these acid-producing, acid-resistant bacterium and microorganisms that enhance biofilm synthesis, aligning perfectly with the tenets of the ecological plaque hypothesis. This discovery indicates potential disturbances in microbial relationships, resulting in ecological imbalances that may facilitate the onset of dental caries. These findings align with those of earlier research studies (Yang et al., 2021).

Within the caries free group, in addition to the close association with *Campylobacter SGB19347* and *Prevotella*, a significant correlation was observed between *Streptococcus rubneri* and *Lachnospiraceae*. *Streptococcus rubneri*, isolated from throat samples of healthy humans in 2013, shared the closest phylogenetic relationship with *Streptococcus australis* (Huch et al., 2013), which inhibited the growth of *Streptococcus mutans* (Huang et al., 2018). The close relationship between *Streptococcus rubneri* and *Lachnospiraceae* may suggest that these two could play a role in inhibiting the development of dental caries. Future *in vitro* experiments may be necessary to validate these interactions.

This study investigated the functional profile of the salivary microbiota in children. Analysis of KEGG pathways uncovered differential expression of K01728 specifically within the severe caries group and K07071 within the caries free group. The pectate lyase (K01728), a glycan metabolism protein (Luis et al., 2018), is linked to pectin metabolism and provides a source of carbon for bacterial growth (Yuan et al., 2019). Within microbial communities, symbiotic bacteria not only produce their own pectate lyases but also induce the production of plant pectate lyases to initiate symbiosis (Hugouvieux-Cotte-Pattat et al., 2014). This process is particularly crucial for vegetarians who rely on microbial consortia for the digestion of pectin (De Angelis et al., 2020). The identification and upregulation of this protein in saliva suggest a similar function in the oral cavity, namely the digestion of oral flora to support bacterial growth in the oral cavity. *Leptotrichia* sp. *oral taxon 498* demonstrates a higher expression of this enzymatic pathway compared to other taxa. Through our correlation analysis, we identified a close relationship between this bacterium and various *Prevotella* species, such as *Prevotella denticola*, *Prevotella veroralis*, and *Prevotella nigrescens*. Previous studies have indicated that *Prevotella* species exhibit a predilection for utilizing pectin (Nograsek et al., 2015); consequently, the relative abundance of *Prevotella* species increases in the presence of pectin (Bang et al., 2018; Yao et al., 2024). Our findings suggest a potential correlation between pectin and dental caries. Conversely, within the healthy group, the differentially expressed KEGG pathways is uncharacterized protein (K07071), which is function unknown and poorly characterized, our result shows that *Haemophilus parainfluenzae* (*p* = 0.0143, FDR = 0.0566) demonstrates a higher expression in this pathway.

Furthermore, we conducted a comparison of the MetaCyc pathways. The Starch Degradation III pathway (PWY-6731), differential elevated in the CS group, is not only critical to the metabolism of dietary starch and sucrose (Hancock et al., 2020), but also to biofilm formation (Liu et al., 2023) by cariogenic bacteria such as *Streptococcus mutans (* Brito et al., 2021). Evidence from studies suggests that the prevention of caries induced by *Streptococcus mutans* could be achieved by inhibiting starch degradation (Culp et al., 2021; Scaffa et al., 2023). The superpathway of menaquinol-9 biosynthesis (PWY-5845) and the superpathway of demethylmenaquinol-9 biosynthesis (PWY-5862) demonstrate varied enhancement in the CF group. Menaquinol-9 convers to demethylmenaquinol-9, and finally produce the menaquinones (Vitamin K2) (Patumcharoenpol et al., 2023), which is pivotal for children’s systemic and oral health, driving a host of physiological functions including blood coagulation, bone mineralization enhancement, and cardiovascular health promotion (Koziol-Kozakowska and Maresz, 2022). Regarding oral health, Vitamin K2 can decelerate the incidence of dental caries not just by slowing demineralization but also by creating a re-mineralization environment for teeth (Southward, 2015). The generation of alkali in oral microbiota predominantly emanates from two principal biochemical routes: the hydrolysis of urea by urease enzymes and the metabolism of arginine facilitated by the arginine deiminase system (ADS) (Liu et al., 2012; Nascimento et al., 2013; Zhu et al., 2022). Superpathway of heme b biosynthesis from uroporphyrinogen-III (PWY0-1415) and urea cycle (PWY-4984) are important pathways for oral bacteria to decompose urea in saliva (Bhushan et al., 2017; Matsumoto et al., 2019). Urea is continuously provided by salivary secretions and gums, and bacterial urease rapidly converts urea into ammonia and carbon dioxide (Mobley and Hausinger, 1989; Righetto et al., 2020). L-citrulline is an intermediate of the L-arginine biosynthetic pathway (Eberhardt et al., 2014), L-citrulline biosynthesis (CITRULBIO-PWY) is closely related to ADS. Primarily, arginine undergoes degradation in the oral cavity through the action of Arginine Deiminase System (ADS), consequently releasing ornithine, ammonia, and CO_2_ (Novak et al., 2016; Mann et al., 2024). Contrary to the urease-mediated decomposition of urea, ADS degrades arginine to supply adenosine triphosphate (ATP) to bacteria (Liu et al., 2012). The subsequent production of alkaline substances through these metabolic pathways acts to inhibit biofilm synthesis.

Our findings identified 16 species, including *Streptococcus mutans*, *Propionibacterium acidifaciens*, and *Prevotella denticola*, were selected based on their strong association with caries severity and high predictive accuracy (AUC = 0.91) in our random forest model. Moving forward, we aim to refine the biomarker panel by reducing the number of species and optimizing the model to enhance clinical feasibility and cost-effectiveness. For example, a subset of 5–10 high-risk species could be used for routine saliva-based screening, enabling early risk stratification and personalized preventive interventions. Future studies will focus on validating this streamlined approach in larger, diverse cohorts.

While this study provides valuable insights into the relationship between the oral microbiome and childhood caries, several limitations should be acknowledged. First, the findings rely solely on salivary samples, which, while convenient and non-invasive, can be influenced by dietary habits, oral hygiene practices, and other transient factors. This may limit the generalizability of our results and underscores the need for complementary sample types, such as dental plaque or tongue coating, in future studies to provide a more comprehensive understanding of the oral microbiome in caries. Second, the cross-sectional design limits our ability to establish causality and observe the dynamic changes in the microbiome over time. Longitudinal studies are needed to better understand the temporal relationships between microbial shifts and caries development. Third, although metagenomic sequencing offers detailed taxonomic and functional information, it does not provide direct evidence of microbial viability or activity within the oral environment. The lack of *in vitro* validation leaves some of the proposed microbial interactions and functional pathways as speculative. We are actively planning follow-up *in vitro* studies to address this gap and further validate our findings.

The findings of this study once more underscore the intricate nature of microbial alterations implicated in dental caries, surpassing initial expectations. Dental caries does not emanate from singular bacteria but rather from shifts in the microbial community structure comprising all oral microorganisms. Investigating the correlation between oral microorganisms and caries necessitates employing sequencing methods with high depth to probe the microbial intricacies accurately, and enhancing the study sample size minimizes errors. Although this study employed metagenomic sequencing to analyze children’s salivary samples, the precision of the results remains unconfirmed through *in vitro* experiments. Moreover, salivary samples are subject to diverse variables such as environmental and dietary influences, contributing to disparities with existing research findings. Consequently, additional *in vitro* experiments are imperative to scrutinize and authenticate the outcomes of this study.

## Conclusion

In conclusion, the role of the oral microbiome in dental caries is multifaceted, with a diverse array of microbial species contributing to its pathogenesis. Our metagenomic analysis has revealed the complex interactions within the oral microbiota and identified potential biomarkers associated with childhood caries. These findings enhance our understanding of the microbial mechanisms underlying the development and progression of dental caries, offering promising avenues for early detection, prevention, and treatment strategies to improve pediatric oral health.

## Data Availability

The data that support the findings of this study have been deposited into CNGB Sequence Archive (CNSA) (Guo et al., 2020) of China National GeneBank DataBase (CNGBdb) (Chen et al., 2020) with accession number CNP0005873.

## Supplementary material

Supplementary Table 1 The children categorized into three groups.

Supplementary Table 2
*Post-hoc* analysis of three groups.

Supplementary Table 3 Recent Research of Dental Caries with Metagenomic Sequencing.

Supplementary Table 4 Presence (Yes/No) of Genera in CF, CL, and CS Groups.
