# Supplementary material for: Novel potential biomarkers for predicting childhood caries via metagenomic analysis
Source: Front Cell Infect Microbiol. 2025 Jun 17;15:1522970. doi: 10.3389/fcimb.2025.1522970 (PMC12209247; doi:10.3389/fcimb.2025.1522970)
Supplement: Supplementary Table 1 — The children categorized into three groups. [file Table1.docx]

**Supplementary information**

| **Supplementary Table 1 \|** **The children categorized into three groups**   \| **category** \| **caries severe**  **(CS, dmft ≥ 6, n = 94)** \| **low-moderate caries**  **(CL, 0 < dmft < 6, n = 99)** \| **caries free**  **(CF, dmft = 0, n = 48)** \| ***p* value** \| \| --- \| --- \| --- \| --- \| --- \| \| Gender (male/ female) \| 50/44 \| 59/40 \| 30/18 \|  \| \| Mouths (mean, SD) \| 98.75,3.47 \| 97.96,3.67 \| 98.71,3.58 \| 0.69 \| \| dmft (mean, SD) \| 8.62,2.60 \| 2.82,1.27 \| 0,0 \| <0.001 \| \| dt (mean, SD) \| 7.8,2.99 \| 2.38,1.44 \| 0,0 \| <0.001 \| \| mt (mean, SD) \| 0.22,0.62 \| 0.15,0.44 \| 0,0 \| 0.022 \| \| ft (mean, SD) \| 0.6,1.43 \| 0.28,0.85 \| 0,0 \| 0.002 \| |
| --- | --- | --- | --- | --- | --- | --- | --- | --- | --- | --- | --- | --- | --- | --- | --- | --- | --- | --- | --- | --- | --- | --- | --- | --- | --- | --- | --- | --- | --- | --- | --- | --- | --- | --- | --- |

| **Supplementary Table 2 \| Post-hoc analysis of three groups** | | | |
| --- | --- | --- | --- |
| **pairs** | **R^2^** | ***p*.value** | ***p*.adjusted** |
| CS vs CL | 0.008153 | 0.086 | 0.086 |
| CS vs CF | 0.040007 | 0.001 | 0.003 |
| CL vs CF | 0.021501 | 0.007 | 0.011 |

**Supplementary Table 3 |Recent Research of Dental Caries with Metagenomic Sequencing**

| **No.** | **Published**  **Date** | **Country** | **DOI** | **Title** | **Sample Type** | **Number**  **of samples** |
| --- | --- | --- | --- | --- | --- | --- |
| 1 | 2017 | Danmark | 10.1038/s41522-017-0031-4 | Metagenomic and metatranscriptomic analysis of saliva reveals disease-associated microbiota in patients with periodontitis and dental caries | Saliva | 30 |
| 2 | 2019 | USA | 10.1080/20002297.2018.1557986 | Metagenome sequencing-based strain-level and functional characterization of supragingival microbiome associated with dental caries in children | Dental Plaque | 30 |
| 3 | 2019 | China | 10.1128/mSystems.00450-19 | Oral Microbiome Alterations Associated with Early Childhood Caries Highlight the Importance of Carbohydrate Metabolic Activities | Saliva | 44 |
| 4 | 2021 | USA | 10.1101/gr.265645.120 | Deep metagenomics examines the oral microbiome during dental caries, revealing novel taxa and co-occurrences with host molecules | Saliva | 47 |
| 5 | 2021 | Turkey | 10.1016/j.archoralbio.2021.105171 | Metagenomic analysis of black-stained plaques in permanent dentition | Dental Plaque | 52 |
| 6 | 2021 | China | 10.1186/s13568-021-01199-4 | Identification of unknown acid-resistant genes of oral microbiotas in patients with dental caries using metagenomics analysis | Saliva | 30 |
| 7 | 2021 | China | 10.3389/fcimb.2021.740981 | Metagenomic Analysis of Dental Plaque on Pit and Fissure Sites With and Without Caries Among Adolescents | Dental Plaque | 40 |
| 8 | 2022 | Spain | 10.1186/s40168-022-01338-4 | Functional changes in the oral microbiome after use of fluoride and arginine containing dentifrices: a metagenomic and metatranscriptomic study | Dental Plaque | 56 |
| 9 | 2022 | China | 10.3760/cma.j.cn112144-20220829-00464 | Saliva microbiota and metabolite in individuals with caries or periodontitis | Saliva | 20 |
| 10 | 2022 | China | 10.3390/nu14183693 | The Oral Microbiome Impacts the Link between Sugar Consumption and Caries: A Preliminary Study | Dental Plaque | 40 |
| 11 | 2023 | China | 10.1080/20002297.2023.2277271 | Multiomics analysis reveals the genetic and metabolic characteristics associated with the low prevalence of dental caries | Saliva | 51 |
| 12 | 2023 | China | 10.3390/microorganisms11102461 | Microbial Relationship of Carious Deciduous Molars and Adjacent First Permanent Molars | Dental Plaque | 42 |
| 13 | 2024 | China | 10.1016/j.jdent.2024.105059 | Metagenomic analysis reveals ecological and functional signatures of oral phageome associated with severe early childhood caries | Dental Plaque | 40 |

| **Supplementary Table 4 \|Presence (Yes/No) of Genera in CF, CL, and CS Groups** | | | |
| --- | --- | --- | --- |
| **Genus** | **CL** | **CS** | **CF** |
| Actinobaculum | yes | yes | yes |
| Actinomyces | yes | yes | yes |
| GGB9835 | yes | yes | yes |
| Pauljensenia | yes | yes | yes |
| Peptidiphaga | yes | yes | yes |
| Schaalia | yes | yes | yes |
| Aeriscardovia | no | yes | no |
| Alloscardovia | yes | yes | no |
| Bifidobacterium | yes | yes | yes |
| Parascardovia | yes | yes | no |
| Scardovia | yes | yes | yes |
| Corynebacterium | yes | yes | yes |
| Lawsonella | no | no | yes |
| Tsukamurella | yes | yes | no |
| Cellulomonas | yes | yes | yes |
| Janibacter | yes | no | no |
| Microbacterium | yes | no | no |
| Kocuria | no | yes | yes |
| Micrococcus | no | yes | no |
| Rothia | yes | yes | yes |
| Isoptericola | yes | yes | no |
| Tropheryma | yes | yes | no |
| Arachnia | yes | yes | yes |
| Cutibacterium | no | yes | no |
| GGB10025 | yes | yes | yes |
| Propionibacterium | yes | yes | yes |
| GGB9369 | yes | no | no |
| Atopobium | yes | yes | no |
| Lancefieldella | yes | yes | yes |
| Olsenella | yes | yes | yes |
| Collinsella | yes | no | no |
| Cryptobacterium | yes | yes | yes |
| Slackia | yes | yes | yes |
| GGB71303 | yes | yes | yes |
| Bacteroidetes_unclassified | yes | yes | yes |
| Bacteroides | yes | yes | no |
| Phocaeicola | yes | yes | yes |
| GGB1088 | yes | yes | yes |
| GGB1431 | yes | yes | no |
| GGB1473 | yes | yes | yes |
| GGB45697 | yes | yes | yes |
| Porphyromonas | yes | yes | yes |
| Alloprevotella | yes | yes | yes |
| GGB1144 | yes | yes | yes |
| GGB1186 | yes | yes | yes |
| GGB33085 | yes | no | no |
| GGB74351 | yes | yes | yes |
| GGB74353 | yes | yes | yes |
| Hallella | yes | yes | yes |
| Prevotella | yes | yes | yes |
| Prevotellaceae_unclassified | yes | yes | yes |
| Alistipes | yes | no | no |
| Tannerella | yes | yes | yes |
| GGB49229 | yes | yes | yes |
| GGB1022 | yes | yes | yes |
| GGB1024 | yes | yes | yes |
| GGB49217 | yes | yes | yes |
| GGB1025 | yes | yes | yes |
| GGB1026 | yes | yes | yes |
| GGB1202 | yes | yes | yes |
| GGB1203 | yes | yes | yes |
| GGB1611 | yes | yes | yes |
| GGB71456 | yes | yes | yes |
| GGB1188 | yes | yes | yes |
| GGB79977 | yes | yes | yes |
| GGB49219 | yes | yes | yes |
| Spirosoma | no | yes | no |
| Capnocytophaga | yes | yes | yes |
| GGB1832 | yes | yes | yes |
| GGB1833 | yes | yes | yes |
| GGB1838 | yes | yes | yes |
| Bergeyella | yes | yes | yes |
| Chryseobacterium | yes | yes | yes |
| Epilithonimonas | no | no | yes |
| GGB1843 | yes | yes | yes |
| GGB1844 | yes | yes | yes |
| GGB1845 | yes | yes | yes |
| Sphingobacterium | no | yes | yes |
| Candidatus_Absconditabacteria_unclassified | yes | yes | yes |
| GGB4936 | yes | yes | yes |
| GGB4937 | yes | yes | yes |
| Candidatus_Gracilibacteria_unclassified | yes | yes | yes |
| GGB4365 | yes | yes | yes |
| GGB12783 | yes | yes | yes |
| GGB70967 | yes | yes | yes |
| GGB12763 | yes | yes | yes |
| Candidatus_Nanoperiomorbus | yes | yes | yes |
| Candidatus_Nanogingivalis | yes | yes | yes |
| GGB12785 | yes | yes | yes |
| GGB12786 | yes | yes | yes |
| Candidatus_Nanosyncoccus | yes | yes | yes |
| Candidatus_Saccharibacteria_unclassified | yes | yes | yes |
| GGB12761 | yes | yes | yes |
| GGB12787 | yes | yes | yes |
| GGB12788 | yes | yes | yes |
| GGB12789 | yes | yes | yes |
| GGB12794 | yes | yes | yes |
| GGB49434 | yes | yes | yes |
| Candidatus_Nanosynbacter | yes | yes | yes |
| Candidatus_Nanosynsacchari | yes | yes | yes |
| Anaerolineaceae_unclassified | yes | yes | yes |
| Deinococcus | yes | yes | no |
| Anoxybacillus | no | no | yes |
| Bacillus | no | yes | yes |
| Geobacillus | yes | no | no |
| Exiguobacterium | no | yes | yes |
| Gemella | yes | yes | yes |
| Macrococcus | no | yes | yes |
| Mammaliicoccus | no | no | yes |
| Staphylococcus | yes | yes | yes |
| Abiotrophia | yes | yes | yes |
| Aerococcus | no | yes | no |
| Dolosigranulum | yes | yes | no |
| Granulicatella | yes | yes | yes |
| GGB10485 | yes | yes | yes |
| GGB15717 | no | yes | no |
| Vagococcus | no | yes | yes |
| Lacticaseibacillus | yes | yes | yes |
| Lactobacillus | yes | yes | yes |
| Lentilactobacillus | no | yes | no |
| Leuconostoc | yes | yes | yes |
| Ligilactobacillus | yes | yes | no |
| Limosilactobacillus | yes | yes | no |
| Pediococcus | no | yes | no |
| Weissella | yes | yes | yes |
| Lactococcus | yes | yes | yes |
| Streptococcus | yes | yes | yes |
| GGB3008 | yes | yes | yes |
| GGB38873 | yes | yes | yes |
| GGB3886 | yes | yes | yes |
| GGB4533 | yes | yes | yes |
| GGB4538 | yes | yes | yes |
| GGB4721 | yes | yes | yes |
| GGB4733 | yes | yes | yes |
| GGB4964 | yes | yes | yes |
| GGB9758 | yes | no | no |
| GGB51647 | yes | no | no |
| GGB49400 | yes | yes | yes |
| GGB4300 | yes | yes | yes |
| GGB3388 | yes | yes | yes |
| GGB3389 | yes | yes | yes |
| GGB3390 | yes | yes | yes |
| Pseudoramibacter | yes | yes | yes |
| Alterileibacterium | yes | no | no |
| Eubacteriales_Family_XIII_Incertae_Sedis_unclassified | yes | yes | yes |
| GGB51110 | yes | no | no |
| Mogibacterium | yes | yes | yes |
| Fenollaria | yes | no | no |
| GGB70946 | yes | yes | yes |
| Levyella | yes | no | no |
| Blautia | yes | no | no |
| Catonella | yes | yes | yes |
| Coprococcus | yes | no | no |
| Dorea | yes | no | no |
| Faecalicatena | yes | no | no |
| Fusicatenibacter | yes | no | no |
| GGB3385 | yes | yes | yes |
| GGB3386 | yes | yes | yes |
| GGB3887 | yes | yes | yes |
| Johnsonella | yes | yes | yes |
| Lachnoanaerobaculum | yes | yes | yes |
| Lachnospira | yes | no | no |
| Lachnospiraceae_unclassified | yes | yes | yes |
| Oribacterium | yes | yes | yes |
| Roseburia | yes | no | no |
| Shuttleworthia | yes | yes | yes |
| Stomatobaculum | yes | yes | yes |
| Faecalibacterium | yes | no | no |
| GGB9635 | yes | no | no |
| Oscillibacter | yes | no | no |
| Oscillospiraceae_unclassified | yes | no | no |
| Ruminococcus | yes | no | no |
| Filifactor | yes | yes | yes |
| GGB4783 | yes | yes | yes |
| GGB4786 | yes | yes | yes |
| GGB50592 | no | no | yes |
| Peptoanaerobacter | yes | yes | yes |
| Peptostreptococcaceae_unclassified | yes | yes | yes |
| Peptostreptococcus | yes | yes | yes |
| Eggerthia | yes | yes | yes |
| Bulleidia | yes | yes | yes |
| Faecalibacillus | yes | no | no |
| Solobacterium | yes | yes | yes |
| Phascolarctobacterium | yes | no | no |
| Centipeda | yes | yes | yes |
| GGB4303 | yes | yes | yes |
| GGB4308 | yes | yes | yes |
| Mitsuokella | yes | yes | yes |
| Pectinatus | no | no | yes |
| Selenomonas | yes | yes | yes |
| Anaeroglobus | yes | yes | yes |
| Dialister | yes | yes | yes |
| GGB4272 | yes | yes | no |
| Megasphaera | yes | yes | yes |
| Veillonella | yes | yes | yes |
| Anaerococcus | yes | no | no |
| Finegoldia | no | yes | no |
| Parvimonas | yes | yes | yes |
| Ezakiella | yes | no | no |
| Fusobacterium | yes | yes | yes |
| GGB56136 | yes | yes | yes |
| GGB4393 | yes | yes | yes |
| GGB4400 | yes | yes | yes |
| GGB49528 | yes | yes | yes |
| GGB71292 | yes | yes | yes |
| Leptotrichia | yes | yes | yes |
| Pseudoleptotrichia | yes | yes | yes |
| Pseudostreptobacillus | yes | yes | yes |
| Caulobacter | yes | no | no |
| GGB7899 | no | yes | no |
| Methylobacterium | yes | yes | yes |
| Phreatobacter | yes | no | no |
| Rhizobium | no | no | yes |
| Paracoccus | yes | no | no |
| Endobacter | yes | no | no |
| Novosphingobium | yes | no | no |
| Sphingomonas | yes | yes | yes |
| Burkholderia | yes | no | no |
| Cupriavidus | yes | no | no |
| Lautropia | yes | yes | yes |
| Ralstonia | yes | no | no |
| Tepidimonas | yes | yes | no |
| Acidovorax | yes | yes | no |
| Caldimonas | yes | no | no |
| Comamonas | no | no | yes |
| Delftia | yes | yes | yes |
| Ottowia | yes | yes | yes |
| Pelomonas | yes | no | no |
| Tibeticola | no | no | yes |
| Zhizhongheella | yes | no | no |
| Eikenella | yes | yes | yes |
| GGB6675 | yes | yes | yes |
| GGB6679 | yes | yes | yes |
| GGB6688 | yes | yes | yes |
| GGB72533 | yes | yes | yes |
| Kingella | yes | yes | yes |
| Neisseria | yes | yes | yes |
| Simonsiella | yes | yes | yes |
| GGB6673 | yes | yes | yes |
| GGB6646 | yes | yes | yes |
| GGB12441 | yes | yes | yes |
| GGB7107 | yes | yes | yes |
| Desulfobulbus | yes | yes | yes |
| Desulfomicrobium | yes | yes | yes |
| Desulfovibrio | yes | yes | yes |
| Campylobacter | yes | yes | yes |
| GGB12440 | yes | yes | yes |
| GGB12443 | yes | yes | yes |
| Cardiobacterium | yes | yes | yes |
| Cronobacter | no | no | yes |
| Enterobacter | no | yes | yes |
| Escherichia | yes | no | yes |
| Klebsiella | yes | yes | yes |
| Phytobacter | no | yes | no |
| Pluralibacter | no | yes | yes |
| Pantoea | no | no | yes |
| Rosenbergiella | no | no | yes |
| Pectobacterium | no | no | yes |
| Acinetobacter | yes | yes | yes |
| GGB72444 | yes | yes | yes |
| Moraxella | yes | yes | yes |
| Psychrobacter | no | yes | yes |
| Carnimonas | no | yes | no |
| Aggregatibacter | yes | yes | yes |
| GGB6813 | yes | yes | yes |
| Haemophilus | yes | yes | yes |
| Pseudomonas | yes | yes | yes |
| Vibrio | no | no | yes |
| Aerosticca | yes | no | no |
| Pseudoxanthomonas | no | yes | no |
| Stenotrophomonas | yes | yes | yes |
| Vulcaniibacterium | yes | no | yes |
| anthomonas | no | yes | yes |
| GGB2671 | yes | yes | yes |
| GGB49499 | yes | yes | yes |
| GGB2665 | yes | yes | yes |
| GGB2666 | yes | yes | yes |
| GGB2672 | yes | yes | yes |
| GGB2674 | yes | yes | yes |
| GGB2676 | yes | yes | yes |
| GGB49504 | yes | yes | yes |
| GGB71076 | yes | yes | yes |
| GGB73508 | yes | yes | yes |
| Treponema | yes | yes | yes |
| Fretibacterium | yes | yes | yes |
| GGB10852 | yes | yes | yes |
| Pyramidobacter | yes | yes | no |
| GGB42243 | yes | no | no |
| GGB4333 | yes | yes | yes |
| Mycoplasma | yes | yes | yes |
| Ureaplasma | no | no | yes |
| Candida | yes | no | no |
